# Data-Driven Pressure Sensor Subset Selection for Long-Distance Water Transfer Pipelines: Q-DEIM Benchmarking with Spatial-Diversity Refinement

**DOI:** 10.3390/s26113601

**Published:** 2026-06-05

**Authors:** Chengkun Liu, Linjie Guan, Siqi Wei

**Affiliations:** 1 Changjiang Survey, Planning, Design and Research Co., Ltd., Wuhan 430010, China; 2 Changjiang Spatial Information Technology Engineering Co., Ltd. (Wuhan), Wuhan 430010, China; 3 Water Resources Information Perception and Big Data Engineering Research Center of Hubei Province, Wuhan 430010, China; 4 Changjiang River Scientific Research Institute of Changjiang Water Resources Commission, Wuhan 430010, China

**Keywords:** data-driven sensor placement, gappy POD, pressure-field reconstruction, Q-DEIM, reduced-order modelling, regime coverage, SCADA, sparse sensing, water transfer pipeline

## Abstract

Long-distance water pipelines are typically instrumented by engineering convention, producing dense and partially redundant networks. Using 7239 hourly snapshots from a 122 km trunk pipeline in northeast China (72 deployed pressure sensors; 60 retained after a >30% missing-rate filter), we ask how few sensors are needed to reconstruct the full pressure field. The field has effective rank 15 at the 99% cumulative-variance level, so the cleaned network is over-sampled by roughly 4×. We benchmark four selection strategies against a random baseline—spatial farthest-point, PCA leverage, Q-DEIM, and a proposed hybrid that adds a soft spatial-diversity penalty to the Q-DEIM residual score—under a uniform Tikhonov-regularised gappy POD reconstruction. Q-DEIM and the hybrid both reach $R^{2}=0.982$ (RMSE 0.96 mH_2_O) with only 15 sensors (75% reduction of the 60-sensor cleaned network); the stricter $R^{2}\geq 0.99$ milestone requires K=26 for Q-DEIM and K=42 for the hybrid. PCA leverage, spatial and random sampling need 19, 32, and 43 sensors for $R^{2}\geq 0.95$. At K=20, the hybrid concedes 0.003 mH_2_O of RMSE for a 3.3× improvement in worst-case fill distance (5.86 km vs. 19.37 km). Regime coverage of the training library is the binding deployment constraint.

## 1. Introduction

Large-scale inter-basin water transfer projects—China’s South-to-North Water Diversion being the most prominent [1]—rely on SCADA networks with pressure sensors distributed along every branch pipeline. Each sensor is a recurring cost: cabling, power, calibration visits, data bandwidth, storage, and the analytics burden of chasing false alarms. Industrial pressure transmitters retail for USD 600–1500 per unit, but the lifetime cost—installation labour, telemetry contracts, and yearly calibration—is typically 3–5× the hardware price [2,3]. For a 72-channel deployment this converts directly into six-figure annual operating expenditure. Yet, the number of sensors is typically fixed during construction by engineering intuition and rarely revisited. Does the network still earn its density once a year of operational data is in hand, or are many of the sensors simply repeating what their neighbours already report?

This paper takes a quantitative answer rather than a qualitative one. We formulate the question as a reconstruction-fidelity benchmark: given any historical record from an over-sampled monitoring network, what is the smallest subset of sensors that still reproduces the full pressure field to within an operational error budget? The framing connects directly to data-driven sparse sensor placement in reduced-order modelling [4,5], but the existing benchmarks come from synthetic CFD or image data rather than real instrumented pipelines, and the algorithms optimise an algebraic reconstruction bound without regard for the geographic placement that operational tasks (leak localisation, zoning, and maintenance routing) demand. The recent data-driven monitoring literature [3,6,7] has extended POD-style reduction in many directions, but the explicit trade-off between reconstruction accuracy and geographic coverage on a real pipeline dataset has, to our knowledge, not been quantified.

The question is not hypothetical. The 122 km trunk line in northeast China that motivates this paper has 72 pressure taps between a source reservoir and two end users, with 22 of them concentrated at a single pump station and the rest distributed along the main pipeline; the record is 10 months at hourly resolution. After dropping 12 sensors with >30% missing data, we work with 60 reliable channels. Principal component analysis of this record finds that 15 modes explain 99% of the variance, so the intrinsic dimension of the pressure field is roughly a quarter of the retained sensor count. That ratio alone does not guarantee the network is removable down to 15 taps: a reduced network must still observe the dominant modes through a well-conditioned measurement matrix. Identifying such a subset is a classical problem in model reduction, known as empirical interpolation [5,8] and gappy POD [9,10] in the fluid mechanics literature, and more recently reframed as data-driven sparse sensor placement [4,11]. In water systems, most existing work on sensor placement addresses leak-location coverage [12,13] rather than field reconstruction, and the few reconstruction-oriented studies in the fluid literature focus on CFD test problems rather than real instrumented pipelines.

The scope here is intentionally focused and application-oriented: four engineered subset-selection strategies, one random baseline, one real operational dataset, and three evaluation criteria. The proposed method is a hybrid of Q-DEIM and a spatial-diversity penalty. Q-DEIM [5] selects sensors by column-pivoted QR on the truncated POD basis and admits a tighter reconstruction-error bound than greedy DEIM [8]; it is among the standard methods in data-driven sensor placement today [4,6]. In this benchmark it gives the lowest reconstruction error for pure reconstruction objectives, but concentrates its picks at hydraulically coupled points—in particular, the pump-station cluster—leaving long unmonitored reaches of pipe. This matters because the same sensor network is routinely used for downstream tasks such as anomaly localisation, which benefit from geographic coverage. Our hybrid adds a penalty proportional to the minimum distance between a candidate and the already-selected set; the weighting is tuned once on training data. At the basis-rank subset size (K=15) the hybrid matches Q-DEIM’s reconstruction RMSE (0.96 mH_2_O; $R^{2}\;=\;0.982$) to three decimal places; as soon as *K* grows to 20, the hybrid’s worst-case fill distance drops from 19.37 km to 5.86 km while RMSE stays within 0.003 mH_2_O of Q-DEIM.

Our main contributions are as follows.

- A reproducible operational benchmark. We compare four sensor-subset strategies on approximately ten months of data from an in-service 122 km trunk water-transfer pipeline (72 deployed sensors; 60 analysed channels after quality control). All methods are evaluated on real SCADA records with the same Tikhonov-regularised gappy-POD reconstruction protocol and the same three metrics (RMSE, $R^{2}$, and fill distance), ensuring direct comparability.
- Evidence on the minimum viable subset and validity boundary. The effective rank of the pressure field (15 modes at 99% cumulative variance) governs the smallest reliable subset when regime coverage is satisfied: both Q-DEIM and the proposed hybrid reach $R^{2}\geq 0.95$ and RMSE ≤1mH_2_O at K=15 (25% of the 60-sensor analysed set). A contiguous time-block stress test then quantifies degradation when this regime-coverage condition is violated.
- A hybrid objective with explicit coverage gains. We augment Q-DEIM residual information with a normalised, unit-free spatial-diversity term controlled by a single scalar. Unlike Clark et al.’s cost-weighted formulation [11], the proposed score acts directly on POD residuals and requires no prior cost model. It reaches the same $R^{2}\;=\;0.95$ milestone as pure Q-DEIM at K=15, while reducing worst-case fill distance at K=20 from 19.37 km to 5.86 km (3.3×) at negligible additional computation.
- A regime-aware decision rule. Around the rank-covering region, both objectives are effectively equivalent; for larger subsets, Q-DEIM prioritises reconstruction error while the hybrid prioritises spatial coverage. A fixed-fidelity localisation proxy (Ψ) summarises this trade-off with a single scalar at each operating point.

The rest of this paper is organised as follows. Section 2 reviews prior work on gappy POD, empirical interpolation, and sensor placement in water networks. Section 3 formalises the reconstruction problem and presents the four ranking strategies. Section 4 describes the dataset, protocol, and results. Section 5 and Section 6 discuss limits and outline next steps.

## 2. Related Work

### 2.1. Reduced-Order Modelling and Gappy POD

Reconstructing a high-dimensional field from partial observations is a mature topic in reduced-order modelling. Proper orthogonal decomposition [14,15,16] extracts a small basis from a library of snapshots and has become the workhorse for fluid, thermal, and plant-wide process data [6,17]. Everson and Sirovich [9] extended POD to the case where each snapshot is only partially observed by introducing the gappy least-squares problem; Willcox [10] applied the idea to unsteady aerodynamic sensing, and Bui-Thanh et al. [18] systematised the choice of basis rank for gappy reconstruction. The approach has since been used for flow-field estimation in experiments, for structural health monitoring [19], and for a range of industrial soft-sensing tasks [20].

### 2.2. Sensor Selection via Empirical Interpolation

Choosing *which* cells to observe so that gappy reconstruction is well conditioned defines the discrete empirical interpolation method (DEIM) [8]. Drmač and Gugercin [5] replaced the original greedy DEIM with column-pivoted QR on the POD basis, a variant (Q-DEIM) that provides a tighter a priori reconstruction-error bound and has become a standard reference method. Manohar et al. [4] unified these ideas into a data-driven sparse sensor placement framework and showed that Q-DEIM-type selection typically outperforms random, heuristic, and convex-relaxation baselines on image and fluid test problems. Alternative formulations route the selection through convex relaxations of the sensor indicator vector [21] or through submodular-optimisation criteria that inherit (1−1/e) approximation guarantees [22].

These methods optimise an algebraic error bound that is agnostic to where the sensors are physically located. On structured grids this is harmless; in a pipeline network, however, the cost of an unmonitored reach is non-trivial, because detection latency for an anomaly at location *x* is at least proportional to the distance from *x* to the nearest observed sensor. Yildirim et al. [23] applied Q-DEIM to ocean-acoustic measurements and noted the same tension; their workaround was to constrain the candidate set to an a priori spatial grid. Clark et al. [11] added explicit cost terms to the greedy selection objective. Papadimitriou [24] approaches a similar spatial concern from an information-gain perspective for structural identification. Our hybrid takes a complementary route: a soft, unit-free spatial-diversity bonus that can be tuned with a single scalar. Whereas Clark et al. [11] require an explicit cost model per candidate sensor and their penalty acts on a greedy information-gain criterion, our penalty is appended directly to the Q-DEIM residual norm, inherits Q-DEIM’s error-bound structure, and introduces no additional problem-specific parameters beyond the diversity weight $\lambda_{s}$.

### 2.3. Sensor Placement in Water Networks

In the water engineering community, the dominant sensor-placement task has been leak localisation rather than full-field reconstruction. Early work casts sensor placement as an integer program over pre-simulated leak or contamination scenarios [12,25], with the objective of minimising the average response time or maximising the separability of event signatures. The Battle of the Water Sensor Networks (BWSN) [25] and the later Battle of the Leakage Detection and Isolation Methods [26] provide common benchmarks for head-to-head comparisons on synthetic water-distribution networks; Hart and Murray [27] review the combinatorial structure of the placement objectives and the approximation algorithms that exploit it. Perelman et al. [13] cast sensor placement for water-quality event detection as a multivariate detection problem, and reviews by Korlapati et al. [2] and Nair et al. [3] survey the subsequent machine-learning efforts.

What is largely missing from this body of work is a reconstruction-oriented analysis: given the historical pressure library, which subset of sensors is sufficient to act as a virtual full field for operational monitoring, and what is the minimum subset size for which this guarantee holds? Wang et al. [28] used all sensors for burst localisation on a long-distance pipeline and did not address subset optimality; Hu et al. [29] build an anomaly-detection pipeline on top of a fixed sensor layout without relating sensor density to reconstruction fidelity. Transient-based leak detection [30,31] relies on a small number of acoustically well-placed sensors but does not provide a recipe for choosing them from a given deployment. To the best of our knowledge, no prior study has simultaneously applied reconstruction-oriented data-driven sensor selection to a real in-service trunk pipeline, quantified the reconstruction–coverage trade-off on operational SCADA data, and identified regime coverage of the training library as the main deployment constraint for this class of methods.

### 2.4. Positioning

We take the Q-DEIM framework from reduced-order modelling, apply it to pressure-field reconstruction on an instrumented water transfer pipeline, and augment it with a spatial penalty that reflects the operational reality of pipeline monitoring. Unlike Casillas et al. and later leak-localisation work, we evaluate on reconstruction fidelity rather than leak-pair separability. Unlike Manohar et al. and Yildirim et al., we use real operational data rather than a synthetic or CFD field. The comparison is purposefully focused: four engineered strategies against a random baseline, one dataset, three evaluation criteria.

Table 1 positions this work against the closest prior benchmarks on the four dimensions that matter for an operational reader: the data domain, whether the spatial layout enters the selection criterion, the form of any spatial constraint, and whether the evaluation is at end-to-end reconstruction (rather than a localisation proxy).

## 3. Materials and Methods

### 3.1. Problem Setting

Let $X\in R^{T\times n}$ be a matrix of hourly pressure snapshots from *n* sensors on a fixed pipeline. A snapshot $x_{t}\in R^{n}$ is a single row of *X*. Given a sensor subset S⊆{1,…,n} of size *K*, we seek a reconstruction operator $R_{S}:R^{K}\to R^{n}$ such that, averaged over a held-out set of snapshots, the error $E∥R_{S}\left(x_{t,S}\right)-x_{t}{∥}_{2}^{2}$ is small.

For a fixed reconstruction family, the optimal subset $S^{*}$ depends on both the distribution of *x* and the family itself. We fix the family to gappy POD and vary *S*; the ranking problem is to choose an ordering π:{1,…,n}→{1,…,n} whose top-*K* prefix is a good subset for every *K* in the sweep.

### 3.2. Gappy POD Reconstruction

**Figure 1:**
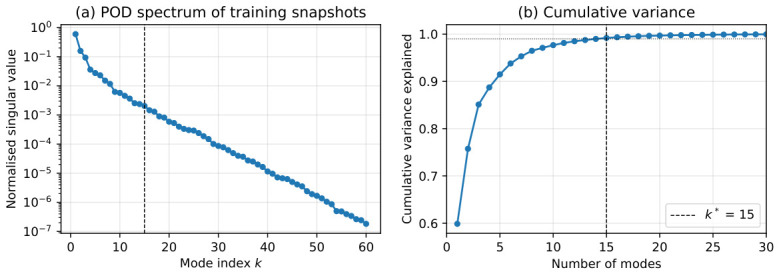
POD spectrum of standardised training snapshots. (**a**) Normalised singular values on log scale; (**b**) cumulative variance explained. The horizontal reference is τ=0.99; the first $k^{*}=15$ modes exceed this threshold.

Let $\mu ,\sigma \in R^{n}$ be the per-sensor mean and standard deviation estimated on training snapshots. We standardise each snapshot as $\tilde{x}=\left(x-\mu \right)/\sigma$ (elementwise). A training matrix ${\tilde{X}}_{\mathrm{train}}$ is decomposed by truncated SVD [14,15,16],

(1) $${\tilde{X}}_{\mathrm{train}}\;\approx \;U\;\Sigma \;V^{⊤},\;V\in R^{n\times k},$$

where *k* is the smallest index for which the cumulative variance ratio $\sum_{i=1}^{k}\sigma_{i}^{2}/\sum_{i}\sigma_{i}^{2}$ exceeds a target threshold τ; here τ=0.99. On our data k=15; see Figure 1. For deployments with *n* in the thousands, the dense SVD can be replaced by a randomised variant [32] without changing the remainder of the pipeline.

Given a subset *S* and a new (standardised) observation ${\tilde{x}}_{S}$ on the observed sensors only, the gappy POD coefficient vector is the Tikhonov-regularised generalisation of the Everson–Sirovich gappy least-squares solution [9,10]:

(2) $$\alpha^{*}\;=\;{\left(V_{S}^{⊤}V_{S}+\lambda I\right)}^{-1}V_{S}^{⊤}\;{\tilde{x}}_{S},$$

where $V_{S}$ denotes the row-restriction of *V* to indices *S*, and $\lambda \;=\;10^{-3}$ is a Tikhonov regulariser [18,33] that keeps the system stable near K≈k. The full reconstruction in physical units is recovered by

(3) $$\hat{x}\;=\;\sigma ⊙\left(V\alpha^{*}\right)+\mu ,$$

with ⊙ denoting elementwise multiplication. The computational cost per snapshot is O(kK+nk) once $V_{S}^{⊤}V_{S}+\lambda I$ has been factored.

### 3.3. Ranking Strategies

Random (baseline).

A uniformly random permutation of sensor indices. We report the mean over 10 seeds.

Spatial farthest-point.

Starting from the sensor closest to the network centroid, at each step we append the sensor maximising the minimum distance to the already-selected set. This encodes only geographic diversity and ignores the POD basis.

PCA leverage.

Sort sensors in decreasing order of their row norm in the truncated POD basis, $\ell_{i}={∥V_{i,:}∥}_{2}^{2}$. A sensor with large leverage participates strongly in the dominant modes and therefore carries information that is hard to recover from the other sensors alone; leverage scores are a standard importance measure in the statistical literature [34,35]. Leverage ranking does not account for how picks interact: two sensors can both have large leverage on the same mode, in which case observing one makes the other redundant.

Q-DEIM.

Apply column-pivoted QR decomposition to $V^{⊤}$—the pivoting strategy of Businger and Golub [36] applied in the Drmač–Gugercin form; the resulting pivot indices $\pi_{1},\ldots ,\pi_{k}$ are the sensor ordering for the first *k* picks [5]. For subsets larger than *k*, we continue with PCA leverage over the unselected sensors.

Hybrid (proposed).

We grow *S* greedily. At iteration *m*, let $R^{\left(m\right)}\in R^{n\times k}$ be the residual of *V* after projecting out the rows corresponding to already-selected sensors, and let $r_{i}={∥R_{i,:}^{\left(m\right)}∥}_{2}$. The hybrid score is

(4) $$s_{i}\;=\;\underset{\mathrm{information}}{\underset{︸}{\frac{r_{i}}{\max_{j}r_{j}}}}\;+\;\lambda_{s}\cdot \underset{\mathrm{spatial}\;\mathrm{diversity}}{\underset{︸}{\frac{\min_{j\in S}{∥p_{i}-p_{j}∥}_{2}}{\max_{j,\ell }{∥p_{j}-p_{\ell }∥}_{2}}}},$$

where $p_{i}\in R^{2}$ is the planar coordinate of sensor *i*, both terms are dimensionless on [0,1], and $\lambda_{s}$ is the diversity weight. After picking $i^{*}=\arg\max_{i}s_{i}$, we update the residual by $R_{i,:}^{\left(m+1\right)}=R_{i,:}^{\left(m\right)}-\langle R_{i,:}^{\left(m\right)},u\rangle u$, where $u=R_{i^{*},:}^{\left(m\right)}/{∥R_{i^{*},:}^{\left(m\right)}∥}_{2}$. The construction reduces to pure Q-DEIM in the limit $\lambda_{s}\;\to \;0$ and to farthest-point sampling as $\lambda_{s}\;\to \;\infty$; for water pipelines we find the reconstruction surface to be remarkably flat in $\lambda_{s}$ on [0.15,0.5] (see Section 4.6), so we fix $\lambda_{s}\;=\;0.35$ for the main results.

Algorithm 1 summarises the procedure.

**Algorithm 1** Hybrid greedy sensor selection**Require:** POD basis $V\in R^{n\times k}$, coordinates ${\left\{p_{i}\right\}}_{i=1}^{n}$, weight $\lambda_{s}$
  1:
S←∅, R←V
  2:
**for** 
m=1,…,n 
**do**
  3:
       **for** each i∉S **do**
  4:
             $r_{i}\leftarrow {∥R_{i,:}∥}_{2}$; $d_{i}\leftarrow \min_{j\in S}{∥p_{i}-p_{j}∥}_{2}$ if S≠∅ else 0
  5:
             $s_{i}\leftarrow r_{i}/\max_{j}r_{j}+\lambda_{s}\;d_{i}/\max_{j,\ell }{∥p_{j}-p_{\ell }∥}_{2}$
  6:
       **end for**
  7:
       $i^{*}\leftarrow \arg\max_{i\notin S}s_{i}$, append to *S*
  8:
       $u\leftarrow R_{i^{*},:}/{∥R_{i^{*},:}∥}_{2}$; update $R\leftarrow R-\left(Ru\right)u^{⊤}$
  9:
**end for**
10:
**return** ordered *S*


### 3.4. Evaluation Protocol

We adopt the snapshot POD evaluation: the *T* snapshots are randomly partitioned 70/30 (fixed seed) into a training library and a held-out test set. The random partition ensures that both splits sample every operating regime, avoiding a known pitfall in chronological partitions of multi-regime pipeline data where the test fold can lie partly outside the training subspace. All quantities—means, standard deviations, POD basis, sensor rankings—are fit on training snapshots only; gappy reconstruction is evaluated on test snapshots.

We report three complementary metrics. Root-mean-square error (RMSE), in physical units (mH_2_O):

(5) $$\mathrm{RMSE}=\sqrt{\frac{1}{T^{\prime }n}\sum_{t,j}{\left(x_{t,j}-{\hat{x}}_{t,j}\right)}^{2}}.$$

Coefficient of determination:

(6) $$R^{2}=1-∥X-\hat{X}{∥}_{F}^{2}/{∥X-\bar{X}∥}_{F}^{2},$$

with $\bar{X}$ the per-sensor mean computed on the test set itself (harder than the train-mean baseline). We also report the train-mean variant ($\bar{X}=\mu$, the deployment-time baseline a practitioner would actually have access to), and find that the two yield identical milestones across all methods on our data; see Section 4.8. Fill distance:

(7) $$\Phi \left(S\right)=\max_{i\in \{1,\ldots ,n\}}\min_{j\in S}{∥p_{i}-p_{j}∥}_{2},$$

the radius of the largest coordinate region that contains no observed sensor (the maximum is taken over all *n* sensors, including those in *S*, since an observed sensor has zero distance to itself and does not inflate the metric), which quantifies spatial coverage independently of reconstruction error. We evaluate it in both Euclidean and along-pipe distance metrics; the two are shown to be interchangeable on this predominantly linear network in Section 4.8.

## 4. Results

### 4.1. Dataset

The data comprise 7239 hourly pressure snapshots (302 days, from 1 November 2024 to 29 August 2025) over 72 sensors along a 122 km trunk water transfer pipeline in Jilin province, northeast China. Pressure measurements are recorded by capacitive ceramic pressure transmitters (accuracy class 0.5, 4–20 mA output, 1-s scan cycle) integrated into the pipeline SCADA system; data are aggregated to hourly means before archiving. Geographic coordinates in WGS-84 decimal degrees are provided per sensor. Pressure ranges from −9.5 to 214.4 mH_2_O across sensors, with per-sensor means spread over an order of magnitude (Table 2); such heterogeneity is typical of pipelines with substantial elevation change between source and users.

We apply three preprocessing steps before analysis. Missing values are imputed by linear interpolation in time, per sensor; gaps longer than a few hours are rare. Sensors with more than 30% missing rate are dropped (12 sensors, leaving 60). Per-sensor standardisation uses the training-fold mean and standard deviation.

### 4.2. POD Spectrum

Figure 1 shows the singular-value spectrum of the standardised training matrix. The spectrum decays rapidly: one mode covers 59.9% of the variance, seven cover 95.3%, and fifteen cover 99.2%. This low-rank structure is the precondition for any reconstruction-based sensor reduction to succeed; it reflects the fact that a steady-state pressure field along a pipeline is largely determined by a small number of upstream boundary conditions and the operational regime of its pumps and valves.

### 4.3. Main Result: Reconstruction vs. Subset Size

**Figure 2:**
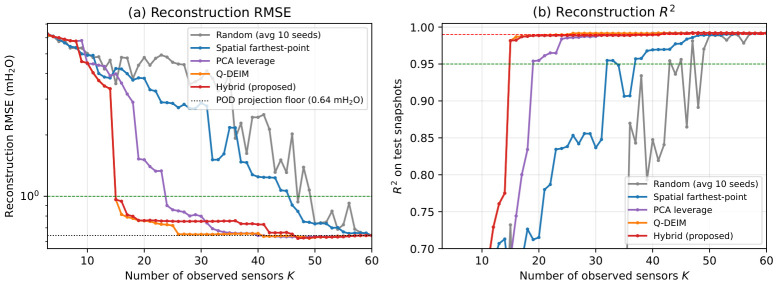
Reconstruction error versus subset size *K* for the five ranking methods. (**a**) RMSE in mH_2_O on log scale; the dashed green line marks the 1 mH_2_O operational target, and the dotted black line is the rank-15 POD projection floor (0.642 mH_2_O). (**b**) Coefficient of determination $R^{2}$ with 0.95 and 0.99 reference lines. Q-DEIM and the hybrid are equivalent up to K=k=15; beyond that, Q-DEIM approaches the projection floor faster.

Figure 2 plots RMSE and $R^{2}$ as a function of *K* for each ranking strategy, with the horizontal $R^{2}\;=\;0.95,\;0.99$ reference lines and the rank-*k* projection floor (0.642  mH_2_O) marked. The curves tell a consistent story. Below K≈k all methods are limited by rank-deficiency and rise sharply; Q-DEIM and the hybrid use the same starting picks and track each other closely. At K=k=15 both reach the $R^{2}\;=\;0.95$ milestone, and RMSE drops below 1 mH_2_O. Beyond K=15 the methods diverge: Q-DEIM descends fastest toward the projection floor, reaching $R^{2}\;=\;0.99$ at K=26, while the hybrid needs K=42 for the same milestone, and PCA leverage K=32. Spatial farthest-point lags substantially because it discards information about which sensors are hydraulically informative (it has no access to *V*); random is worst.

Table 3 summarises the subset-size milestones. A consistent result is that both Q-DEIM and the hybrid reach $R^{2}\geq 0.95$ with only 15 observed sensors out of 60—a 75% reduction. At a practical 1 mH_2_O RMSE tolerance, the same pattern holds. PCA leverage is competitive (19 and 24 sensors for the two milestones, respectively), which makes it a reasonable zero-effort baseline. Spatial sampling and random require 2–3 times more sensors to reach the same accuracy.

### 4.4. Geographic Coverage

**Figure 3:**
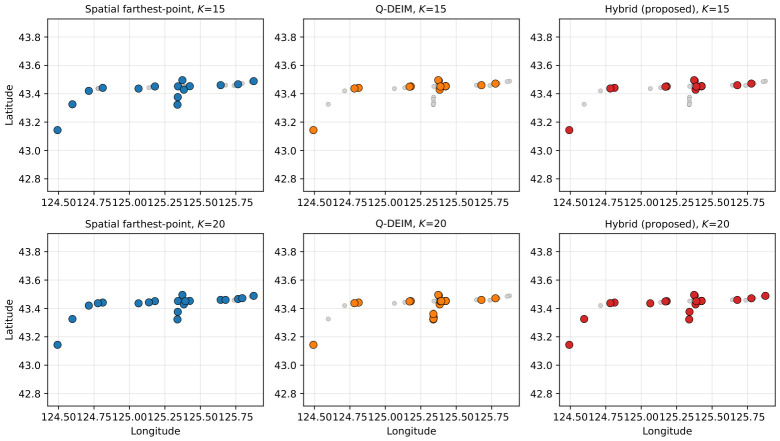
Selected sensors plotted on the pipeline map at K=15 (**top row**) and K=20 (**bottom row**). Selected sensors are coloured; the unselected are light grey. Q-DEIM concentrates picks at the hydraulically coupled pump station; the hybrid redistributes redundant picks toward peripheral branches.

The reconstruction-error advantage of Q-DEIM comes at a spatial cost. Figure 3 shows the first K=15 and K=20 selections on the pipeline map. Q-DEIM concentrates picks at the 22-sensor pump-station co-location (the leading PCA modes are dominated by it) and leaves a 19.37 km stretch without coverage at K=15. At this subset size the hybrid selects an alternative subset with comparable information content, and both methods share the same fill distance of 19.37 km; as *K* increases to 20, the spatial penalty takes effect and the hybrid’s fill distance falls to 5.86 km. Spatial sampling alone gives the lowest fill distance by construction, at the cost of ignoring which sensors are actually informative.

**Figure 4:**
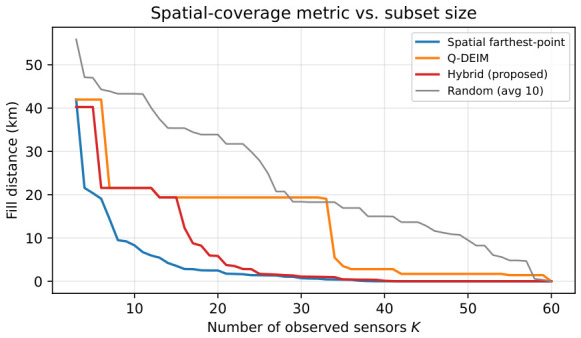
Fill distance (radius of the largest coordinate region with no observed sensor) versus subset size. Spatial farthest-point is lowest by construction. Q-DEIM lags because its picks are algebraically but not geographically spread. The hybrid closes most of the gap.

Figure 4 gives the fill-distance curve across the full *K* sweep. Pure spatial sampling is, unsurprisingly, best on this metric alone; the hybrid sits between spatial and Q-DEIM but tracks spatial closely beyond K=20, while Q-DEIM alone catches up only near K=30. At K=15—the minimum viable reduction—the hybrid’s fill distance matches Q-DEIM’s exactly (19.37 km): the first fifteen picks are too informationally loaded to be displaced by the spatial term. Once a few slots are free of basis-rank pressure, the spatial penalty takes over: at K=20 the hybrid’s fill distance drops to 5.86 km, a 3.3× reduction, while the corresponding Q-DEIM value stays at 19.37 km until K≥30.

### 4.5. Per-Sensor Errors and a Qualitative Example

**Figure 5:**
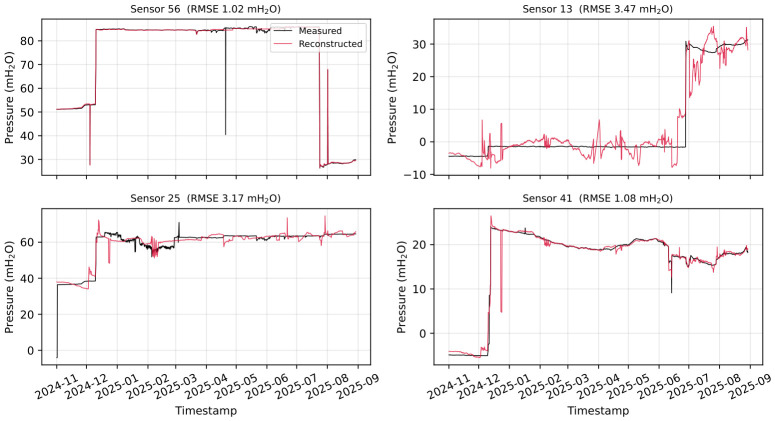
Reconstructed (red) vs. measured (black) pressure at four unobserved sensors, K=15 hybrid ranking. Sensors were chosen by test-set variance to cover a range of operational dynamics.

**Figure 6:**
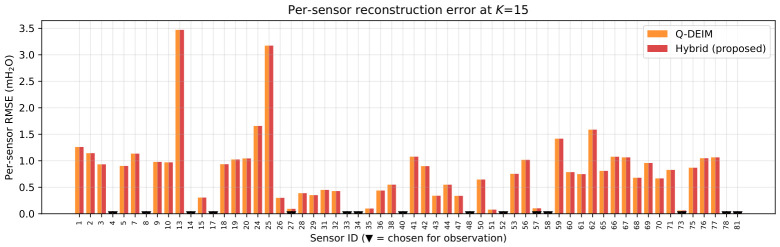
Per-sensor reconstruction RMSE at K=15 for Q-DEIM (orange) and the hybrid (red). Downward triangles on the baseline mark the sensors chosen for observation. Errors are dominated by a handful of high-variance sensors regardless of ranking method.

Figure 5 shows reconstructed vs. measured pressure at four unobserved sensors selected by test-set variance, for K=15 and the hybrid ranking. The reconstruction tracks both the low-frequency envelope (operational regime) and the short pressure excursions at the hour scale. Per-sensor reconstruction RMSEs are reported in Figure 6, with downward triangles marking chosen sensors. At K=15 the hybrid is within ±0.5 mH_2_O of Q-DEIM on every sensor; the largest residuals concentrate on the same handful of non-stationary sensors regardless of ranking.

### 4.6. Ablation on the Spatial-Diversity Weight

We swept $\lambda_{s}\in \left\{0.15,\;0.25,\;0.35,\;0.50\right\}$ and found that reconstruction RMSE at the $R^{2}\;\geq \;0.95$ milestone is unaffected, while fill distance at K=20 shifts only at the decimal level (all four values yield 5.86 km on our data). This suggests the hybrid’s utility comes from its structural form—adding any spatial-diversity term to a Q-DEIM-like objective—rather than from fine tuning; $\lambda_{s}$ can be set by operational preference. A practitioner who values geographic coverage above marginal reconstruction fidelity should choose $\lambda_{s}$ near the upper end of this range; the opposite priority should drive it toward zero, recovering pure Q-DEIM.

### 4.7. Sensitivity to the Imputation Rule

The 12.7% overall missing-data rate is filled by linear interpolation in time, per sensor, before downstream analysis. To check robustness we repeated the headline experiments with three alternative rules: forward fill (replace each gap with the last observed value), per-sensor median fill, and per-sensor mean fill. Table 4 reports the resulting $R^{2}$ at K∈{10,15,20,30} for Q-DEIM and the hybrid, together with the basis rank chosen at the 99% cumulative-variance threshold under each rule.

Two observations follow. First, the headline K=15 result is specific to linear interpolation: the cruder rules add step or constant-region artefacts that consume an extra one or two POD modes, pushing the milestone out to K=17 or K=20. Second, by K=20 the four imputation rules agree to within 0.011 in $R^{2}$, so the sensor-subset ranking itself is robust; only the very tight regime near $K\;\approx \;\;k^{*}$ is sensitive to imputation quality. The choice of linear interpolation is defensible on physical grounds: pressure transients are continuous in time, gaps in the record are short (median sensor missing rate 2.34%), and hold-last-value imputation creates artificial plateaus that are inconsistent with the dynamics of a pumped pipeline.

### 4.8. Along-Pipe Distance and $R^{2}$-Baseline Robustness

Section 3 computed fill distance with Euclidean (straight-line) distance, but a fluid in a pipeline travels along the pipeline route, not as the crow flies. We checked the discrepancy by projecting each sensor onto the principal axis of the network coordinate scatter and integrating the haversine distance between consecutive unique locations. The pipeline along-route length is approximately 166 km compared with a 122 km straight-line bounding-box diagonal (a roughly 27% detour). The Pearson correlation between Euclidean and along-pipe pairwise distances across all sensor pairs is r=0.958 (Spearman 0.951), confirming that on this predominantly linear network the two metrics are interchangeable to high precision. Repeating the fill-distance analysis at the headline subset sizes confirms the conclusion: at K=20 the hybrid’s along-pipe fill distance is 5.87 km (vs. 5.86 km Euclidean), and Q-DEIM’s is 19.94 km (vs. 19.37 km Euclidean). The 3.3× improvement in coverage reported earlier is unchanged.

We also verified that switching the $R^{2}$ baseline from the test-set mean to the more deployment-realistic training-set mean (the only baseline a practitioner could compute online) preserves every milestone in Table 3 to the integer *K*. This is a consequence of the random-partition design: train and test snapshots are drawn from the same distribution by construction, and the two means differ in the third decimal place per sensor. Section 5 returns to the implications of this design choice for chronologically partitioned data, where the equivalence no longer holds.

### 4.9. Regime Coverage: Validity Boundary and Deployment Implications

The random 70/30 split used for the main benchmark ensures that both folds draw from the same empirical distribution of operating regimes, which is the correct protocol for quantifying the information content of a given library. However, in a live deployment the model is trained once and then applied as new data arrive; if the pipeline enters a regime not present in the training library, the reconstruction basis is misspecified. To quantify this boundary, we ran a five-fold contiguous time-block evaluation: the 302-day record is divided into five consecutive blocks, each block serving as the test fold while the remaining four form the training fold. This setting is deliberately adversarial—each fold boundary may coincide with a regime transition—and provides an upper bound on the degradation that can occur when regime coverage fails.

Table 5 contrasts the two protocols. Under regime-matched (random) splitting, Q-DEIM and the hybrid reach $R^{2}\;\geq \;0.95$ at K=15 as reported above. Under the contiguous stress test, no method exceeds $R^{2}\;=\;0.31$ in any fold; fold-mean $R^{2}$ values are negative, indicating that reconstruction error exceeds the per-sensor variance baseline when the basis is extrapolated. These numbers are not a failure of the selection strategy—all four methods degrade equally—but a diagnostic of the regime-coverage requirement: the gappy-POD framework is a *compression* of a given library, not an *extrapolation* beyond it. The practical implication is that deployment should verify training coverage before activating sparse observation, monitor online reconstruction residuals and trigger retraining when they exceed the acceptance threshold, and fall back to denser observation when an unseen regime is detected. Section 5 elaborates a concrete deployment checklist.

### 4.10. Downstream Localisation Proxy at Fixed Fidelity Regime

The fill-distance curves indicate better geographic coverage for the hybrid, but downstream localisation tasks also require acceptable reconstruction fidelity. We therefore define a simple proxy at fixed subset size:

(8) $$\Psi \left(S\right)=\left\{\begin{array}{cc} 1/\Phi (S), & R^{2}\left(S\right)\geq 0.95,\\ 0, & R^{2}\left(S\right)<0.95, \end{array}\right.$$

where Φ(S) is fill distance in km. The indicator threshold avoids rewarding geographically spread but low-fidelity subsets.

At K=20, Q-DEIM and the hybrid have nearly identical reconstruction fidelity ($R^{2}\approx 0.989$), but very different coverage: $\Phi_{Q-\mathrm{DEIM}}=19.37$ km versus $\Phi_{\mathrm{Hybrid}}=5.86$ km. The resulting proxy is 0.0516 for Q-DEIM and 0.1708 for the hybrid, i.e., a 3.31× improvement. For reference, spatial farthest-point has the smallest fill distance but fails the $R^{2}\geq 0.95$ condition at K=20, so its proxy is zero.

### 4.11. Implementation and Runtime

All experiments were run on a single commodity laptop CPU in Python 3 with NumPy and SciPy. The dominant cost is the initial SVD of the 60-dimensional training matrix (∼5000 snapshots), which completes in well under one second; for larger deployments a randomised SVD [32] could be swapped in without changing the subsequent pipeline. A full *K*-sweep over {3,…,60} for all five ranking methods, including ten random-seed repetitions for the baseline, completes in under four seconds from data load to CSV export. The online reconstruction cost per incoming snapshot is O(kK+nk) after one Cholesky factorisation of $V_{S}^{⊤}V_{S}+\lambda I$, which is itself $O(k^{3})$; in absolute terms this is under 100 μs per snapshot at k=15. The approach is therefore well within the budget of a real-time SCADA update loop, and the full benchmark can be re-run whenever new months of data accumulate.

## 5. Discussion

Why the minimum is 15 sensors.

The 15-sensor lower bound mirrors the effective rank of the training snapshot matrix at the 99% cumulative-variance threshold. This relationship is expected: below K=k the measurement matrix, $V_{S}$ is tall-and-thin but also rank-limited by the number of observations, so the coefficient estimate in (2) becomes ill-conditioned, and the Tikhonov regularisation pays a bias price. Reducing the sensor count below the effective rank requires either a richer basis (e.g., time-dependent POD; nonlinear manifold models) or an acceptance of lower reconstruction fidelity. A pipeline operator who insists on K<15 should first ask whether the operational envelope covered by the training library is truly low-rank.

Why Q-DEIM concentrates its picks.

The first Q-DEIM pick is the sensor with the largest row norm in the truncated POD basis; on our data this is the pump-station co-location, which captures the dominant operational regime. Subsequent picks are chosen to keep the restricted basis well-conditioned, which favours sensors whose signal is near-orthogonal to the already-selected set. Because the pump-station sensors span a rich subspace of hydraulic modes, the next few picks tend to be drawn from the same station. The spatial-diversity term in the hybrid breaks this tie at a small but real cost in conditioning, which is why the hybrid’s $R^{2}\;\to \;0.99$ milestone moves from K=26 to K=42.

Physical interpretation of the headline figures.

Figure 5 shows that the hybrid subset reproduces both the slowly varying envelope that reflects operational regime (pumping schedule; downstream demand) and the higher-frequency excursions on the order of one to a few hours that correspond to valve actuations and load redistribution. Two qualitative features of the per-sensor errors in Figure 6 are worth noting. First, the small handful of sensors with residual RMSE above 2 mH_2_O are concentrated at the hydraulic boundaries—the source-reservoir tap and the pump-station outlet—where the dynamics include rapid events (valve switching) that lie outside the slow modes captured by the rank-15 basis. Second, sensors located mid-pipe show near-floor residuals because their local pressure is well predicted as a smooth function of the upstream and downstream boundary states. This pattern mirrors the standard separation between boundary and interior cells in reduced-order modelling of advection-diffusion fields and supports a physically motivated reading of the POD modes as the dominant pressure gradients propagating along the trunk pipe.

Measurement uncertainty.

The errors reported in this paper are type A in the sense of the BIPM Guide to the Expression of Uncertainty in Measurement [37]: statistical residuals against the held-out test snapshots. Industrial pressure transmitters used on water transfer pipelines have a typical type B accuracy of ±0.075% to ±0.25% of full scale [2], which for a 200 mH_2_O operational range translates to ±0.15 to ±0.50 mH_2_O of instrument uncertainty. The headline reconstruction RMSE of 0.96 mH_2_O at K=15 is therefore within roughly a factor of two of the sensors’ own intrinsic noise floor: pushing *K* above 20 buys statistical RMSE below the instrument calibration uncertainty, at which point further sensor reduction is constrained by hardware rather than by the reconstruction model.

Economic implications.

On the population of long-distance water transfer pipelines, industrial pressure transmitters of the precision class used here retail for USD 600–1500 per unit, with lifetime cost typically 3–5× the hardware price once installation labour, telemetry contracts, and yearly calibration are included [2,3]. Removing 45 of the 60 reliable sensors retained after preprocessing therefore translates to a direct capital-expenditure saving of order USD 30–70 k for a single trunk line, plus the proportional operating-expenditure saving on the lifetime. For a regional water utility operating several trunk lines, the benefit scales linearly. The hybrid’s improved geographic coverage is a second-order economic factor: an unmonitored 19 km stretch raises the expected detection latency for a leak by an order of magnitude relative to a 5 km stretch, and shorter latency translates into smaller spilled volumes and lower emergency-response costs.

Validity boundary and generalisability.

Several properties of the dataset bound the scope of the reported reduction ratios. The pipeline is predominantly linear: pressure at any tap is largely determined by a small set of upstream boundary conditions and a smooth elevation profile, which produces the low effective rank of 15. Branching networks with hydraulically decoupled loops would likely have higher effective rank, a smaller achievable reduction ratio, and greater benefit from the hybrid’s spatial term at lower *K*. Most critically, the training library must cover every operational regime seen at deployment. A Gaussian-mixture analysis of the same time series separates it into roughly a dozen distinct operating states; the random-split protocol samples all states in both folds, satisfying this condition. The contiguous time-block stress test (Section 4.9) deliberately violates it and confirms that all methods degrade similarly when coverage fails, identifying regime coverage as the binding constraint rather than any property of the selection algorithm itself. In addition, the gappy-POD model assumes Gaussian-like residual structure; pressure transients and hydraulic shocks lie outside this model and should be handled by a dedicated anomaly-detection layer operating in parallel with the reconstructed field.

Relation to leak localisation.

A sparser sensor set can still support leak localisation if the reconstructed field is accurate at the sensor-free reaches. The hybrid’s improved fill distance is therefore of direct downstream value: at K=20, the worst unmonitored gap of 5.86 km is within the length scale at which transient-based leak detection methods remain sensitive [30]. A dedicated study is needed to quantify the localisation-fidelity trade-off; we view it as natural follow-up work.

Deployment checklist.

The contiguous stress-test results motivate a concrete operational workflow. We recommend the following guardrails for rolling out sensor reduction on a live pipeline:

- **Regime audit before activation.** Before switching to the reduced sensor set, verify that the training library spans all known pump-scheduling modes and seasonal demand states. A clustering check (e.g., Gaussian mixture model on the snapshot matrix) can confirm that the deployment period does not introduce new cluster centroids.
- **Hold-out acceptance gate.** Evaluate reconstruction accuracy on a recent hold-out window (≥two weeks) using the acceptance thresholds $R^{2}\;\geq \;0.95$ and RMSE ≤1 mH_2_O before activating sparse observation.
- **Online residual monitoring.** After activation, compute per-snapshot reconstruction residuals in real time. A sustained breach of the RMSE threshold on any rolling 24 h window should trigger an alert.
- **Drift-triggered retraining.** When residuals exceed the threshold, refit the POD basis and re-run the sensor-selection sweep on the updated library. Because the benchmark completes in under four seconds, this can be scheduled as a nightly cron job or triggered on demand.
- **Fallback policy.** Maintain a protocol that temporarily restores observation to the full deployed sensor set when an unseen regime is detected, providing ground-truth data to expand the training library.

In combination, these guardrails convert the one-off optimisation into a maintainable monitoring policy that remains valid as the pipeline ages and its operating envelope evolves.

Limitations.

Several limitations remain. We used a single in-service dataset, so replication on pipelines with different topologies (branching networks, booster stations, and variable-demand profiles) is needed to assess how far the rank-15 structure and the 75% reduction ratio generalise. The reconstruction family is linear (Tikhonov-regularised gappy POD); nonlinear alternatives—graph-signal interpolation, kernel-based methods, or neural-operator surrogates—may improve accuracy in transient-dominated regimes at the cost of increased model complexity. The hybrid’s spatial penalty uses Euclidean planar distance as a proxy for hydraulic proximity. For the present pipeline—a largely straight trunk line with modest lateral branches—the correlation between Euclidean and along-pipe distance is high, but a topological pipeline model would provide a more faithful metric for branched or looped networks. Finally, milestone *K* values are reported as point estimates from a single fixed train/test split; bootstrap confidence intervals over repeated splits would strengthen the statistical interpretation of milestone comparisons.

## 6. Conclusions

We benchmarked four sensor-subset selection strategies—spatial farthest-point, PCA leverage, Q-DEIM, and a proposed hybrid—on approximately ten months of operational pressure records from a 122 km in-service water transfer pipeline (72 deployed sensors; 60 retained after quality control), using a uniform Tikhonov-regularised gappy-POD reconstruction protocol and three complementary metrics. Across this benchmark, we find that when the training library covers the operational regime, the effective rank of the pressure field (15 modes at 99% cumulative variance) is both a sufficient statistic for the minimum viable subset size and a necessary condition for reconstruction fidelity to hold.

Under regime-matched evaluation, Q-DEIM and the proposed hybrid both achieve $R^{2}\;\geq \;0.95$ and RMSE ≤1 mH_2_O with only 15 sensors—a 75% reduction relative to the 60-sensor analysed set. Beyond the rank-covering regime (K>15), pure Q-DEIM approaches the POD projection floor fastest; the hybrid trades a small amount of reconstruction accuracy for a 3.3× improvement in worst-case geographic coverage (fill distance 5.86 km vs. 19.37 km at K=20), a property of direct value for downstream tasks such as leak localisation. A fixed-fidelity proxy Ψ quantifies this trade-off in a single scalar, with the hybrid scoring 3.31× higher than Q-DEIM at K=20.

A contiguous time-block stress test identifies regime coverage as the binding deployment constraint: all methods degrade equally when the training library fails to cover the deployment regime, confirming that the coverage requirement is a property of the gappy-POD framework itself rather than of any particular selection strategy. A deployment checklist—regime audit, hold-out gate, online residual monitoring, drift-triggered retraining, and fallback policy—converts the one-off optimisation into a maintainable monitoring policy. The full benchmark runs in under 4 s on a commodity laptop, making scheduled retraining practical as new data accumulate.

In practice, pure Q-DEIM is preferable when reconstruction fidelity is the primary objective, whereas the hybrid is more suitable when geographic coverage is an explicit operational constraint. Across the entire *K* sweep, both methods outperform heuristic and random selection by a wide margin.

Future work includes extending the spatial penalty to use along-pipe distance (requiring a topological pipeline model) to better reflect hydraulic proximity in branched networks, quantifying whether the hybrid’s improved fill distance translates to better anomaly-localisation accuracy on the reconstructed field, and replacing the linear POD basis with a nonlinear surrogate—graph-signal interpolation or a neural operator—to extend validity into transient-dominated operating regimes.

## Figures and Tables

**Table 1:** Positioning of our benchmark against the closest prior work. “Soft penalty” refers to the unit-free spatial-diversity term in Equation (4), which contrasts with the hard candidate-set restriction of Yildirim et al. [23] and the cost-weighted greedy of Clark et al. [11].

Work	Data Domain	Spatial in Objective	Constraint Form	Reconstruction Metric
Drmač & Gugercin [5]	synthetic	no	—	yes (RMSE)
Manohar et al. [4]	CFD/image	no	—	yes (RMSE)
Yildirim et al. [23]	ocean acoustics	yes	hard grid	yes (RMSE)
Clark et al. [11]	synthetic	via cost	cost weight	yes (RMSE)
Casillas et al. [12]	water network	yes	topology	no (leak coverage)
This work	water pipeline	yes	soft penalty	yes (RMSE, $R^{2}$, Φ)

**Table 2:** Dataset summary. Co-location indicates sensors sharing the same geographic coordinate (same pump or valve station, redundantly instrumented). Effective rank is the number of POD modes required to capture ≥99% of the standardised training snapshot variance.

Quantity	Value
Raw sensors	72
Unique geographic locations	47
Largest co-location (pump station)	22 sensors
Time range	1 November 2024 to 29 August 2025
Sampling interval	1 h
Snapshots (*T*)	7239
Missing rate, overall	12.66%
Missing rate, per-sensor median	2.34%
Sensors retained after >30% missing filter	60
Network bounding-box diagonal	122 km
Effective rank at 99% variance	15

**Table 3:** Smallest subset size *K* at which each method first reaches the column milestone. Lower is better. RMSE is in mH_2_O. The minimum feasible RMSE is the rank-15 POD projection floor at 0.642 mH_2_O; no method is listed for a 0.5 mH_2_O milestone because the floor exceeds it.

Method	$K\;:\;R^{2}\;\geq \;0.90$	$K\;:\;R^{2}\;\geq \;0.95$	$K\;:\;R^{2}\;\geq \;0.99$	K:RMSE≤2	K:RMSE≤1
Random (avg 10)	38	43	54	36	47
Spatial farthest-point	32	32	53	32	46
PCA leverage	19	19	32	19	24
Q-DEIM	15	15	26	15	15
Hybrid (proposed, $\lambda_{s}\;=\;0.35$)	15	15	42	15	15

**Table 4:** Sensitivity of reconstruction $R^{2}$ (train-mean baseline) to the imputation rule. Linear interpolation preserves short-range temporal correlation and gives the cleanest 15-mode basis; cruder rules introduce step or constant-region artefacts that inflate the basis rank to 16–17 and push the K=15 milestone out, although all rules converge at K=20 to within 0.011 in $R^{2}$.

Imputation	Method	K=10	K=15	K=20	K=30	Basis Rank
Linear (default)	Q-DEIM	0.598	0.982	0.989	0.992	15
	Hybrid	0.598	0.982	0.989	0.989	15
Forward fill	Q-DEIM	0.588	0.717	0.977	0.982	16
	Hybrid	0.588	0.717	0.978	0.983	16
Median	Q-DEIM	0.298	0.759	0.981	0.988	17
	Hybrid	0.339	0.759	0.981	0.982	17
Mean	Q-DEIM	0.573	0.737	0.983	0.984	17
	Hybrid	0.585	0.737	0.979	0.983	17

**Table 5:** Regime-matched (random 70/30) versus regime-adversarial (contiguous time-block) evaluation. The contiguous protocol quantifies the degradation when the training library does not cover the deployment regime; equal collapse across all methods confirms this is a coverage effect, not a selection artefact. Fold statistics are over five contiguous blocks. $R^{2}@15$ denotes test $R^{2}$ at K=15.

Method	Baseline $K\;:\;R^{2}\;\geq \;0.95$	Fold $R^{2}@15$ (Mean ± Std)	Best Fold $R^{2}$ (Max)	Folds with $R^{2}\;\geq \;0.95$
Spatial farthest-point	32	−2.12±3.20	0.313	0/5
PCA leverage	19	−2.54±3.48	0.270	0/5
Q-DEIM	15	−3.00±4.26	0.300	0/5
Hybrid (proposed)	15	−2.82±4.54	0.285	0/5

## Data Availability

Raw SCADA records are subject to confidentiality restrictions imposed by the pipeline operator and are therefore not publicly released. A reproducibility package containing all benchmark scripts, plotting scripts, and derived CSV artefacts (rmse_vs_k.csv, rankings.csv, summary.json, and supplementary split/proxy tables) is available from the corresponding author upon reasonable request. The benchmark is implemented in Python 3.x with NumPy, SciPy, and Matplotlib; the package is sufficient to reproduce all figures and tables in this manuscript from the derived artefacts without access to the raw records.

## Abbreviations

The following abbreviations are used in this manuscript:

POD	Proper Orthogonal Decomposition
DEIM	Discrete Empirical Interpolation Method
Q-DEIM	QR-based Discrete Empirical Interpolation Method
SVD	Singular Value Decomposition
PCA	Principal Component Analysis
RMSE	Root Mean Square Error
SCADA	Supervisory Control and Data Acquisition
